# 4-Meth­oxy-2-{(*E*)-[(thio­phen-2-yl)methyl­imino]­meth­yl}phenol

**DOI:** 10.1107/S1600536812036586

**Published:** 2012-08-31

**Authors:** Esen Nur Kantar, Yavuz Köysal, Mustafa Macit, Ebru Er, Mustafa Serkan Soylu

**Affiliations:** a Department of Physics, Faculty of Arts and Sciences, Ondokuz Mayıs University, TR-55139 Samsun, Turkey; b Yesilyurt Demir Celik Vocational School, Ondokuz Mayıs University, TR-55139 Samsun, Turkey; c Department of Chemistry, Faculty of Arts and Sciences, Ondokuz Mayıs University, Kurupelit, 55139 Samsun, Turkey; d Department of Physics, Faculty of Arts and Sciences, Giresun University, Giresun, Turkey

## Abstract

The title Schiff base, C_13_H_13_NO_2_S, adopts the phenol–imine tautomeric form and reveals an intra­molecular O—H⋯N hydrogen bond involving the hy­droxy group and the imino N atom, forming an *S*(6) ring. The mol­ecule is highly twisted with respect to the central imine group, which is reflected in the dihedral angle of 67.83 (10)° formed by the thienyl and phenol rings. The crystal packing is characterized by weak C—H⋯O and C—H⋯π inter­actions.

## Related literature

Schiff bases of salicyl­aldehyde may exhibit thermochromism or photochromism, depending on the planarity or non-planarity, respectively, of the mol­ecule, see: Amimoto & Kawato (2005 ▶); Schmidt & Cohen (1964 ▶). For a related structure, see: Kantar *et al.* (2012 ▶). For hydrogen-bond motifs, see: Bernstein *et al.* (1995 ▶)
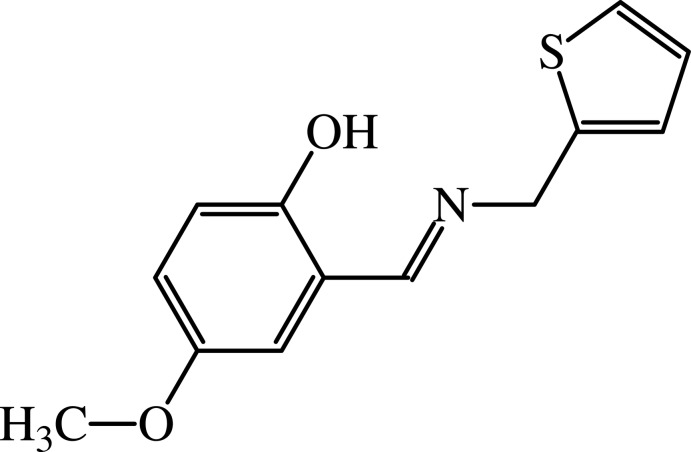


## Experimental

### 

#### Crystal data

C_13_H_13_NO_2_S
*M* *_r_* = 247.30
Monoclinic, 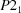

*a* = 5.6325 (3) Å
*b* = 8.1666 (3) Å
*c* = 13.4836 (6) Å
β = 96.798 (4)°
*V* = 615.86 (5) Å^3^
*Z* = 2
Mo *K*α radiation
μ = 0.25 mm^−1^
*T* = 293 K
0.20 × 0.15 × 0.10 mm

#### Data collection

Oxford Diffraction SuperNova (single source at offset) Eos diffractometer
Absorption correction: multi-scan (*CrysAlis PRO*; Oxford Diffraction, 2007 ▶) *T*_min_ = 0.951, *T*_max_ = 0.975
2221 measured reflections
1809 independent reflections
1472 reflections with *I* > 2σ(*I*)
*R*_int_ = 0.015

#### Refinement

*R*[*F* ^2^ > 2σ(*F* ^2^)] = 0.047
*wR*(*F* ^2^) = 0.109
*S* = 1.06
1809 reflections
159 parameters
2 restraints
H atoms treated by a mixture of independent and constrained refinement
Δρ_max_ = 0.16 e Å^−3^
Δρ_min_ = −0.21 e Å^−3^
Absolute structure: Flack (1983 ▶), 640 Friedel pairs
Flack parameter: 0.04 (13)

### 

Data collection: *CrysAlis PRO* (Oxford Diffraction, 2007 ▶); cell refinement: *CrysAlis PRO*; data reduction: *CrysAlis PRO*; program(s) used to solve structure: *SHELXS97* (Sheldrick, 2008 ▶); program(s) used to refine structure: *SHELXL97* (Sheldrick, 2008 ▶); molecular graphics: *ORTEP-3 for Windows* (Farrugia, 1997 ▶); software used to prepare material for publication: *WinGX* (Farrugia, 1999 ▶).

## Supplementary Material

Crystal structure: contains datablock(s) I, global. DOI: 10.1107/S1600536812036586/zq2177sup1.cif

Structure factors: contains datablock(s) I. DOI: 10.1107/S1600536812036586/zq2177Isup2.hkl

Supplementary material file. DOI: 10.1107/S1600536812036586/zq2177Isup3.cml

Additional supplementary materials:  crystallographic information; 3D view; checkCIF report

## Figures and Tables

**Table 1:** Hydrogen-bond geometry (Å, °) *Cg*1 is the centroid of the C1–C6 ring.

*D*—H⋯*A*	*D*—H	H⋯*A*	*D*⋯*A*	*D*—H⋯*A*
O1—H1⋯N1	1.10 (6)	1.63 (7)	2.616 (4)	147 (5)
C8—H8*A*⋯O2^i^	0.97	2.77	3.592 (4)	143
C2—H2⋯*Cg*1^ii^	0.93	3.00	3.631 (4)	127

Symmetry codes: (i) 
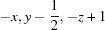
; (ii) 
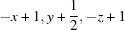
.

**Fig. 1.**
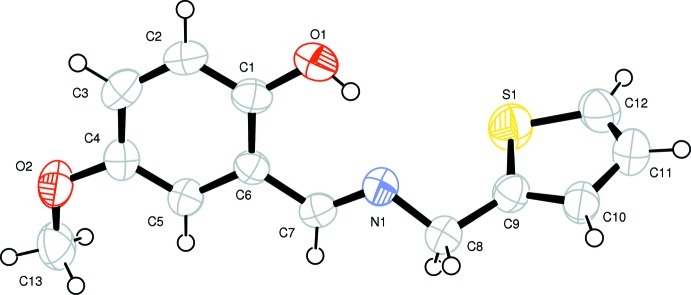
A view of the title compound with the atom-numbering scheme and 50% probability displacement ellipsoids.

**Fig. 2.**
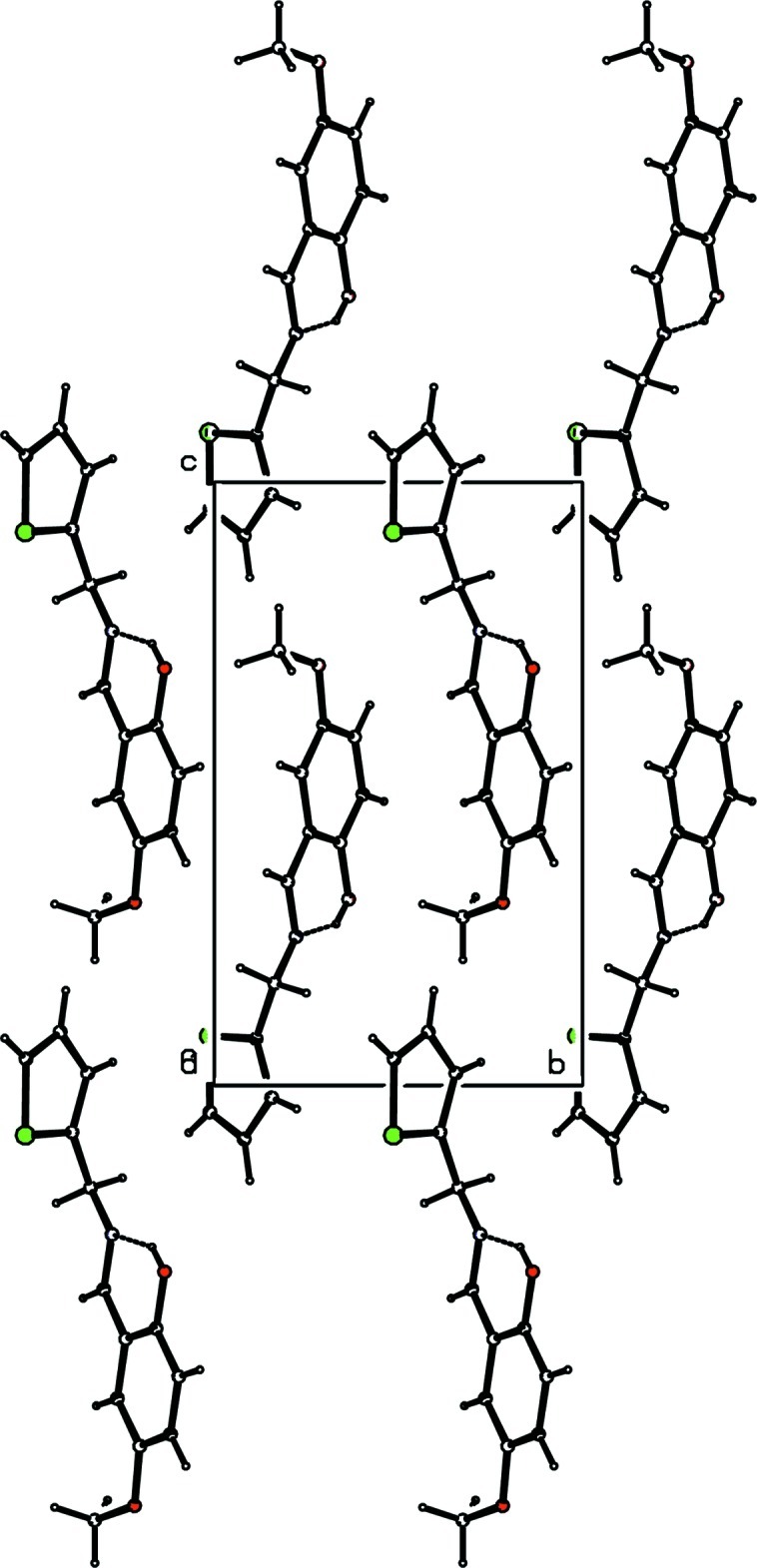
Packing diagram of the title compound along the a axis.

## supplementary crystallographic information

### Comment

The title Schiff base, C_13_H_13_NO_2_S, adopts in the crystal structure the phenol-imine tautomeric form and reveals an intramolecular O—H···N hydrogen bond between the hydroxy O atom and the imino N atom (Kantar *et al.*, 2012) generating a nearly planar six-membered ring, a S(6) ring motif according to Bernstein *et al.* (1995).

It is known that Schiff bases of salicylaldehyde may exhibit thermochromism or photochromism, depending on planarity or non-planarity of the molecule (Schmidt & Cohen, 1964; Amimoto & Kawato, 2005). The X-ray diffraction study of the title compound shows that the molecule is highly twisted with respect to the central imine group, which is reflected in the dihedral angle of 67.83 (10)° formed by the thienyl and phenol rings.

The crystal packing is characterized by weak C—H···O (see Table 1) and C—H···π interactions: the distance between C2—H2 and Cg1*^ii^* (C1-C6 ring) is 3.00 Å [symmetry code: *ii* = 1-*x*, 0.5+*y*, 1-*z*].

### Experimental

The title compound was prepared by refluxing a mixture of a solution containing 2-hydroxy-5-methoxybenzaldehyde (15.22 mg, 0.1 mmol) in ethanol (20 ml) and a solution containing 2-thiophenemethylamine (11.32 mg, 0.1 mmol) in ethanol (20 ml). The reaction mixture was stirred for 5 h under reflux. Single crystals of the title compound suitable for X-ray analysis were obtained by slow evaporation of the ethanol solution (yield 64%; m.p. 353–355 K).

### Refinement

The structure was solved by direct methods and refined by full-matrix least-square techniques. All hydrogen positions, except H1 which was located in a difference Fourier map and freely refined), were calculated after each cycle of refinement using a riding model, with C—H = 0.93 Å and *U*_iso_(H) = 1.2*U*_eq_(C) for aromatic H atoms, with C—H = 0.97 Å and *U*_iso_(H) = 1.2*U*_eq_(C) for methine H atoms, and with C—H = 0.96 Å and *U*_iso_(H) = 1.5*U*_eq_(C) for methyl H atoms. The Flack *x* parameter was refined to 0.04 (13) based on 640 Friedel pairs.

### Crystal data

**Table:**

C_13_H_13_NO_2_S	*F*(000) = 260
*M**_r_* = 247.30	*D*_x_ = 1.334 Mg m^−^^3^
Monoclinic, *P*2_1_	Mo *K*α radiation, λ = 0.71073 Å
Hall symbol: P 2yb	Cell parameters from 958 reflections
*a* = 5.6325 (3) Å	θ = 3.6–28.7°
*b* = 8.1666 (3) Å	µ = 0.25 mm^−^^1^
*c* = 13.4836 (6) Å	*T* = 293 K
β = 96.798 (4)°	Block, yellow
*V* = 615.86 (5) Å^3^	0.20 × 0.15 × 0.10 mm
*Z* = 2	

### Data collection

**Table:**

Oxford Diffraction SuperNova (single source at offset) Eos diffractometer	1809 independent reflections
Radiation source: fine-focus sealed tube	1472 reflections with *I* > 2σ(*I*)
Graphite monochromator	*R*_int_ = 0.015
Detector resolution: 16.0454 pixels mm^-1^	θ_max_ = 25.0°, θ_min_ = 3.6°
ω scans	*h* = −3→6
Absorption correction: multi-scan (*CrysAlis PRO*; Oxford Diffraction, 2007)	*k* = −9→9
*T*_min_ = 0.951, *T*_max_ = 0.975	*l* = −15→16
2221 measured reflections	

### Refinement

**Table:**

Refinement on *F*^2^	Secondary atom site location: difference Fourier map
Least-squares matrix: full	Hydrogen site location: inferred from neighbouring sites
*R*[*F*^2^ > 2σ(*F*^2^)] = 0.047	H atoms treated by a mixture of independent and constrained refinement
*wR*(*F*^2^) = 0.109	*w* = 1/[σ^2^(*F*_o_^2^) + (0.0439*P*)^2^] where *P* = (*F*_o_^2^ + 2*F*_c_^2^)/3
*S* = 1.06	(Δ/σ)_max_ = 0.009
1809 reflections	Δρ_max_ = 0.16 e Å^−^^3^
159 parameters	Δρ_min_ = −0.21 e Å^−^^3^
2 restraints	Absolute structure: Flack (1983), 640 Friedel pairs
Primary atom site location: structure-invariant direct methods	Flack parameter: 0.04 (13)

### Special details

**Table:**

Geometry. All e.s.d.'s (except the e.s.d. in the dihedral angle between two l.s. planes) are estimated using the full covariance matrix. The cell e.s.d.'s are taken into account individually in the estimation of e.s.d.'s in distances, angles and torsion angles; correlations between e.s.d.'s in cell parameters are only used when they are defined by crystal symmetry. An approximate (isotropic) treatment of cell e.s.d.'s is used for estimating e.s.d.'s involving l.s. planes.
Refinement. Refinement of *F*^2^ against ALL reflections. The weighted *R*-factor *wR* and goodness of fit *S* are based on *F*^2^, conventional *R*-factors *R* are based on *F*, with *F* set to zero for negative *F*^2^. The threshold expression of *F*^2^ > σ(*F*^2^) is used only for calculating *R*-factors(gt) *etc*. and is not relevant to the choice of reflections for refinement. *R*-factors based on *F*^2^ are statistically about twice as large as those based on *F*, and *R*- factors based on ALL data will be even larger.

### Fractional atomic coordinates and isotropic or equivalent isotropic displacement parameters (Å^2^)

**Table:**

	*x*	*y*	*z*	*U*_iso_*/*U*_eq_	
C1	0.3932 (6)	0.8415 (4)	0.5974 (3)	0.0481 (9)	
C2	0.4982 (7)	0.9033 (4)	0.5174 (3)	0.0551 (10)	
H2	0.6417	0.9604	0.5289	0.066*	
C3	0.3927 (7)	0.8808 (4)	0.4220 (3)	0.0562 (10)	
H3	0.4663	0.9221	0.3691	0.067*	
C4	0.1768 (7)	0.7970 (4)	0.4023 (3)	0.0500 (9)	
C5	0.0698 (7)	0.7362 (4)	0.4812 (2)	0.0449 (9)	
H5	−0.0738	0.6795	0.4689	0.054*	
C6	0.1748 (6)	0.7589 (4)	0.5800 (2)	0.0415 (8)	
C7	0.0515 (7)	0.6993 (4)	0.6618 (3)	0.0440 (9)	
H7	−0.0921	0.6433	0.6472	0.053*	
C8	−0.0063 (7)	0.6629 (5)	0.8305 (3)	0.0586 (11)	
H8A	−0.1025	0.5702	0.8050	0.070*	
H8B	−0.1139	0.7489	0.8468	0.070*	
C9	0.1489 (7)	0.6132 (4)	0.9223 (3)	0.0506 (9)	
C10	0.1217 (7)	0.6557 (5)	1.0203 (3)	0.0562 (10)	
H10	0.0014	0.7232	1.0389	0.067*	
C11	0.3051 (8)	0.5809 (6)	1.0888 (3)	0.0726 (13)	
H11	0.3178	0.5960	1.1576	0.087*	
C12	0.4554 (8)	0.4878 (7)	1.0440 (3)	0.0725 (12)	
H12	0.5821	0.4302	1.0781	0.087*	
C13	−0.1056 (9)	0.6748 (6)	0.2794 (3)	0.0737 (13)	
H13A	−0.1514	0.6756	0.2085	0.111*	
H13B	−0.2383	0.7091	0.3128	0.111*	
H13C	−0.0587	0.5661	0.3004	0.111*	
N1	0.1345 (6)	0.7215 (4)	0.7533 (2)	0.0528 (8)	
O1	0.5060 (5)	0.8643 (3)	0.6917 (2)	0.0659 (8)	
O2	0.0879 (6)	0.7829 (3)	0.30331 (18)	0.0652 (8)	
S1	0.3895 (2)	0.48631 (16)	0.91768 (8)	0.0757 (4)	
H1	0.386 (11)	0.814 (9)	0.743 (4)	0.17 (2)*	

### Atomic displacement parameters (Å^2^)

**Table:**

	*U*^11^	*U*^22^	*U*^33^	*U*^12^	*U*^13^	*U*^23^
C1	0.040 (2)	0.046 (2)	0.058 (2)	0.0053 (19)	0.0061 (18)	−0.0043 (18)
C2	0.041 (2)	0.056 (2)	0.069 (3)	−0.006 (2)	0.009 (2)	0.0000 (19)
C3	0.060 (3)	0.049 (2)	0.063 (3)	0.001 (2)	0.022 (2)	0.0059 (19)
C4	0.061 (3)	0.044 (2)	0.045 (2)	0.004 (2)	0.0071 (19)	−0.0015 (16)
C5	0.050 (2)	0.0383 (18)	0.047 (2)	−0.0017 (18)	0.0080 (17)	0.0000 (16)
C6	0.0394 (19)	0.0395 (18)	0.045 (2)	0.0054 (18)	0.0042 (16)	−0.0009 (15)
C7	0.042 (2)	0.0389 (19)	0.051 (2)	0.0017 (18)	0.0079 (18)	0.0005 (16)
C8	0.052 (2)	0.078 (3)	0.047 (2)	0.002 (2)	0.0097 (19)	0.003 (2)
C9	0.054 (2)	0.049 (2)	0.050 (2)	0.001 (2)	0.0098 (19)	0.0036 (16)
C10	0.060 (2)	0.064 (2)	0.045 (2)	0.004 (2)	0.0089 (19)	0.0020 (18)
C11	0.078 (3)	0.091 (3)	0.050 (3)	−0.005 (3)	0.011 (2)	0.003 (2)
C12	0.069 (3)	0.080 (3)	0.066 (3)	−0.001 (3)	−0.001 (2)	0.017 (3)
C13	0.087 (3)	0.086 (3)	0.045 (2)	−0.006 (3)	0.000 (2)	−0.008 (2)
N1	0.0490 (19)	0.063 (2)	0.0468 (18)	0.0014 (17)	0.0085 (15)	0.0032 (15)
O1	0.0475 (16)	0.085 (2)	0.0622 (19)	−0.0050 (16)	−0.0050 (14)	−0.0040 (15)
O2	0.085 (2)	0.0653 (17)	0.0457 (16)	−0.0113 (17)	0.0085 (15)	0.0064 (13)
S1	0.0763 (8)	0.0815 (8)	0.0709 (7)	0.0178 (7)	0.0153 (6)	−0.0011 (6)

### Geometric parameters (Å, º)

**Table:**

C1—O1	1.365 (4)	C8—H8A	0.9700
C1—C2	1.385 (5)	C8—H8B	0.9700
C1—C6	1.398 (5)	C9—C10	1.392 (5)
C2—C3	1.364 (5)	C9—S1	1.713 (4)
C2—H2	0.9300	C10—C11	1.438 (5)
C3—C4	1.393 (5)	C10—H10	0.9300
C3—H3	0.9300	C11—C12	1.334 (6)
C4—O2	1.374 (4)	C11—H11	0.9300
C4—C5	1.375 (5)	C12—S1	1.699 (4)
C5—C6	1.404 (4)	C12—H12	0.9300
C5—H5	0.9300	C13—O2	1.410 (5)
C6—C7	1.456 (4)	C13—H13A	0.9600
C7—N1	1.279 (4)	C13—H13B	0.9600
C7—H7	0.9300	C13—H13C	0.9600
C8—N1	1.462 (4)	O1—H1	1.10 (6)
C8—C9	1.486 (5)		
			
O1—C1—C2	118.7 (3)	C9—C8—H8B	109.3
O1—C1—C6	121.7 (3)	H8A—C8—H8B	108.0
C2—C1—C6	119.6 (3)	C10—C9—C8	127.1 (4)
C3—C2—C1	120.4 (4)	C10—C9—S1	111.2 (3)
C3—C2—H2	119.8	C8—C9—S1	121.7 (3)
C1—C2—H2	119.8	C9—C10—C11	110.6 (4)
C2—C3—C4	121.2 (4)	C9—C10—H10	124.7
C2—C3—H3	119.4	C11—C10—H10	124.7
C4—C3—H3	119.4	C12—C11—C10	113.4 (4)
O2—C4—C5	125.3 (4)	C12—C11—H11	123.3
O2—C4—C3	115.8 (3)	C10—C11—H11	123.3
C5—C4—C3	118.8 (3)	C11—C12—S1	112.5 (3)
C4—C5—C6	120.8 (3)	C11—C12—H12	123.7
C4—C5—H5	119.6	S1—C12—H12	123.7
C6—C5—H5	119.6	O2—C13—H13A	109.5
C5—C6—C1	119.1 (3)	O2—C13—H13B	109.5
C5—C6—C7	119.4 (3)	H13A—C13—H13B	109.5
C1—C6—C7	121.5 (3)	O2—C13—H13C	109.5
N1—C7—C6	122.1 (3)	H13A—C13—H13C	109.5
N1—C7—H7	119.0	H13B—C13—H13C	109.5
C6—C7—H7	119.0	C7—N1—C8	118.2 (3)
N1—C8—C9	111.6 (3)	C1—O1—H1	106 (3)
N1—C8—H8A	109.3	C4—O2—C13	117.3 (3)
C9—C8—H8A	109.3	C12—S1—C9	92.2 (2)
N1—C8—H8B	109.3		
			
O1—C1—C2—C3	179.0 (3)	C1—C6—C7—N1	1.1 (5)
C6—C1—C2—C3	−1.5 (5)	N1—C8—C9—C10	132.6 (4)
C1—C2—C3—C4	0.6 (5)	N1—C8—C9—S1	−49.0 (5)
C2—C3—C4—O2	179.7 (3)	C8—C9—C10—C11	178.8 (3)
C2—C3—C4—C5	−0.1 (5)	S1—C9—C10—C11	0.3 (4)
O2—C4—C5—C6	−179.3 (3)	C9—C10—C11—C12	−0.8 (6)
C3—C4—C5—C6	0.4 (5)	C10—C11—C12—S1	0.9 (5)
C4—C5—C6—C1	−1.3 (5)	C6—C7—N1—C8	177.7 (3)
C4—C5—C6—C7	177.3 (3)	C9—C8—N1—C7	149.1 (3)
O1—C1—C6—C5	−178.7 (3)	C5—C4—O2—C13	−11.4 (5)
C2—C1—C6—C5	1.8 (5)	C3—C4—O2—C13	168.9 (3)
O1—C1—C6—C7	2.7 (5)	C11—C12—S1—C9	−0.6 (4)
C2—C1—C6—C7	−176.8 (3)	C10—C9—S1—C12	0.1 (3)
C5—C6—C7—N1	−177.5 (3)	C8—C9—S1—C12	−178.5 (3)

### Hydrogen-bond geometry (Å, º)

Cg1 is the centroid of the C1–C6 ring.

**Table:**

*D*—H···*A*	*D*—H	H···*A*	*D*···*A*	*D*—H···*A*
O1—H1···N1	1.10 (6)	1.63 (7)	2.616 (4)	147 (5)
C8—H8*A*···O2^i^	0.97	2.77	3.592 (4)	143
C2—H2···*Cg*1^ii^	0.93	3.00	3.631 (4)	127

Symmetry codes: (i) −*x*, *y*−1/2, −*z*+1; (ii) −*x*+1, *y*+1/2, −*z*+1.

## References

[bb1] Amimoto, K. & Kawato, T. (2005). *J. Photochem. Photobiol. C*, **6**, 207–226.

[bb2] Bernstein, J., Davis, R. E., Shimoni, L. & Chang, N.-L. (1995). *Angew. Chem. Int. Ed. Engl.* **34**, 1555–1573.

[bb3] Farrugia, L. J. (1997). *J. Appl. Cryst.* **30**, 565.

[bb4] Farrugia, L. J. (1999). *J. Appl. Cryst.* **32**, 837–838.

[bb5] Flack, H. D. (1983). *Acta Cryst.* A**39**, 876–881.

[bb6] Kantar, E. N., Köysal, Y., Gümüş, S., Ağar, E. & Soylu, M. S. (2012). *Acta Cryst.* E**68**, o1587. 10.1107/S160053681201882X PMC3379200 22719398

[bb7] Oxford Diffraction (2007). *CrysAlis PRO* Oxford Diffraction Ltd, Abingdon, England.

[bb8] Schmidt, G. M. J. & Cohen, M. D. (1964). *J. Chem. Soc.* pp. 1996–2000.

[bb9] Sheldrick, G. M. (2008). *Acta Cryst.* A**64**, 112–122. 10.1107/S0108767307043930 18156677

